# Unraveling the Stratification of an Iron-Oxidizing Microbial Mat by Metatranscriptomics

**DOI:** 10.1371/journal.pone.0102561

**Published:** 2014-07-17

**Authors:** Achim Quaiser, Xavier Bodi, Alexis Dufresne, Delphine Naquin, André-Jean Francez, Alexandra Dheilly, Sophie Coudouel, Mathieu Pedrot, Philippe Vandenkoornhuyse

**Affiliations:** 1 Université de Rennes 1, CNRS UMR6553 EcoBio, Rennes, France; 2 CNRS FRC3115 Centre de Recherches de Gif-sur-Yvette, Gif sur Yvette, France; 3 Université de Rennes 1, CNRS UMS3343 OSUR, Plateforme génomique environnementale et fonctionnelle, Rennes, France; 4 Université de Rennes 1, CNRS UMR6118 Géosciences, Rennes, France; Missouri University of Science and Technology, United States of America

## Abstract

A metatranscriptomic approach was used to study community gene expression in a naturally occurring iron-rich microbial mat. Total microbial community RNA was reversely transcribed and sequenced by pyrosequencing. Characterization of expressed gene sequences provided accurate and detailed information of the composition of the transcriptionally active community and revealed phylogenetic and functional stratifications within the mat. Comparison of 16S rRNA reads and delineation of OTUs showed significantly lower values of metatranscriptomic-based richness and diversity in the upper parts of the mat than in the deeper regions. Taxonomic affiliation of rRNA sequences and mRNA genome recruitments indicated that iron-oxidizing bacteria affiliated to the genus *Leptothrix*, dominated the community in the upper layers of the mat. Surprisingly, type I methanotrophs contributed to the majority of the sequences in the deep layers of the mat. Analysis of mRNA expression patterns showed that genes encoding the three subunits of the particulate methane monooxygenase (*pmo*CAB) were the most highly expressed in our dataset. These results provide strong hints that iron-oxidation and methane-oxidation occur simultaneously in microbial mats and that both groups of microorganisms are major players in the functioning of this ecosystem.

## Introduction

A fundamental objective of microbial ecology is to identify patterns of composition, abundance and activity of microorganisms along environmental gradients. Knowledge of these patterns is essential to understand which processes control microbial community structure and how communities adapt to environmental changes. In this context, microbial mats represent excellent models for unveiling the relationships between community structure and ecosystem functioning. Microbial mats typically thrive in transition zones with steep environmental gradients where they may form some of the most impressive structures built up by microorganisms [1].

Iron-rich microbial mats occur worldwide in circumneutral pH freshwater habitats where anoxic, Fe(II)-rich groundwaters reach surface and encounter oxygenated conditions [2]–[7]. Neutrophilic iron-oxidizers Bacteria (FeOB) affiliated to the *Betaproteobacteria Leptothrix* (*Burkholderiales*) and *Gallionella/Sideroxydans* (*Nitrosomonadales*) flourish at these oxic-anoxic interfaces where they use Fe(II) as an electron donor to produce energy and sustain growth e.g. [5]. Accretion of extracellular structures produced by *Leptothrix* (sheaths) and *Gallionella* (stalks), that are encrusted with iron hydroxides, results in the formation of slimy layers of orange aggregates characteristic of the iron-rich mats [1],[6],[8],[9]. The mats can reach up to several tens of centimeters of thickness and provide a habitat for numerous other microorganisms. Indeed, in addition to the lithotrophic mat-building FeOB, culture-independent studies have shown the existence of a large diversity of Bacteria in the mat, including phyla of uncultured Bacteria [4],[6],[7],[10]. However, whether these microorganisms are endemic to the iron-rich mat community remains to be determined.

The developments of high-throughput sequencing techniques have dramatically expanded our knowledge of taxonomic diversity as well as protein-coding gene repertoire in natural communities, thus revolutionizing the field of microbial ecology. In this context, random sequencing of the pool of transcripts extracted from environmental samples, named metatranscriptomics hereafter, constitutes an effective technique to target both community structure and the expressed genes that acquaint with community functions carried out at the time of sampling [11]–[13]. In this study, we report for the first time the use of metatranscriptomics for characterizing community gene expression profiles of a typical iron-rich microbial mat. We address the hypothesis of a strong vertical community structuring within the microbial mat. We provide evidence that even if taxonomic composition does not change significantly with depth, there is a clear phylogenetic and functional stratification between the surface of the mat and the subjacent deeper regions. In particular, we show that methane-oxidizing microorganisms likely dominate the community at depth.

## Results

### Microbial mat sampling characteristics and pyrosequencing read statistics

Measurements of physico-chemical parameters showed that the iron concentrations were high in all samples and mainly present as ferrous iron, ranging from 1.6–12.64 mg/l (see Materials and Methods, Table S1), indicating favorable conditions for the oxidation of iron by microorganisms [14],[15]. Methane concentrations were measured over a period from January 2013 to July 2013 (Figure S1 in Supporting Information S1). The methane concentration was 1–2 orders of magnitude higher in the microbial mat than in the surrounding water flows without microbial mat observation (t-test *p* = 0.0033). While the differences between surface and deep samples were low, in five out of six measurements the methane concentrations were higher in deep samples. A novel specific nucleic acid treatment pipeline was applied for total RNA extraction, purification, cDNA synthesis and pyrosequencing (see Materials and Methods). After size and quality trimming, 2,082,630 cDNA sequences with an average size of 449 bp were obtained from the five samples and three replicates in two pyrosequencing runs (Table 1). One replicate from location D3 failed in sequencing and was excluded from the analyses. On average, 83.35% of the reads matched to rRNA sequences (28.78% SSU and 54.57% LSU), 7.91% had matches with the NCBI nr database whereas 8.74% of the sequences had no hit.

**Table 1:** Characteristics of the metatranscriptomic libraries used for comparative diversity analysis.

	Surface 1	Surface 2	Depth 1	Depth 2	Depth 3	Combined
N° of fragments	408,993	281,431	330,304	395,633	666,269	2.08 Mio.
Average size (bp)	434	456	441	442	471	449
Total nucleotides (Mbp)	177.4	128.3	145.5	174.8	314.0	934
GC content	50.6%	50.2%	51.0%	52.8%	50.3%	
SSU rRNA matches	29.03%	30.18%	28.12%	22.92%	33.67%	28.78%
LSU rRNA matches	53.84%	59.58%	57.52%	49.07%	52.82%	54.57%
Matches against nr	8.35%	5.13%	7.24%	13.66%	5.16%	7.91%
No matches	8.79%	5.10%	7.12%	14.35%	8.35%	8.74%
Comment	Triplicate	Triplicate	Triplicate	Triplicate	Duplicate	

Average values from replicates are shown.

### Taxonomic affiliation of rRNA transcribed sequences

**Figure 1:**
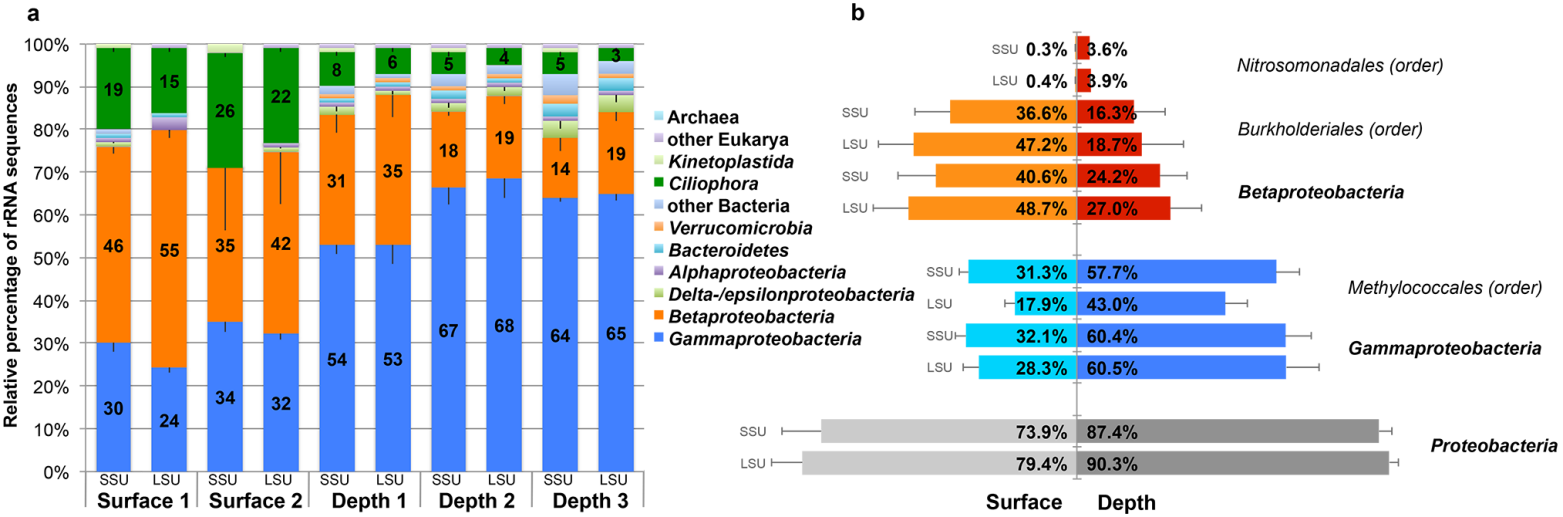
Relative abundance and distribution of microbial taxa in the SSU and LSU rRNA transcript libraries. The rRNA reads from each triplicate are combined and average values shown. (a) Comparison of the five samples at the phylum level. (b) Comparison of the combined two surfaces (S1 and S2) and the subjacent depth (D1 and D2) samples at the phylum, class and order level. Error bars indicate the standard deviations among the replicates.

The taxonomic affiliations of the SSU and LSU rRNA sequences were determined by comparison to the SILVA non-redundant rRNA database with BLASTn [16],[17]. Processing the rRNA BLAST hits with MEGAN [18] revealed that the mat was composed of 34 microbial phyla including 21 bacterial, 2 archaeal and 11 eukaryal phyla. However, 96.3% of the sequences were affiliated to only 7 and 1 bacterial and eukaryal phyla, respectively (Figure 1a). The most abundant SSU rRNA reads in the different samples were affiliated to *Gammaproteobacteria* and *Betaproteobacteria*, which represented on average 53.0% and 25.3% of the assigned SSU rRNA reads respectively, whereas 10.9% were affiliated to the eukaryal phylum Ciliophora (Alveolata). Most of the eukaryal sequences were related to typical freshwater grazers such as *Tetrahymena* species (Figure S2 in Supporting Information S1) which could feed on the microbial community thriving in the mat [19]. In addition to these 3 dominant phyla, 6.19% of the total SSU rRNA reads, on average, were assigned to *Alpha*-, *Delta*- and *Epsilonproteobacteria* as well as to Bacteroidetes and Verrucomicrobia. Archaeal transcripts represented only about 0.2% of the sequences. Interestingly, more than 96% of the *Gammaproteobacteria* were affiliated to *Methylococcales* (Figure 1b), which consist exclusively of methylotrophs and type I methanotrophs. Conversely, only 0.065% of the total SSU rRNAs sequences were affiliated to families within the *Alphaproteobacteria* containing type II methanotrophs, which suggests that these microorganisms likely played a minor role in the community functioning. As expected, 66% to 90% of the *Betaproteobacteria* were affiliated to *Burkholderiales* and *Nitrosomonadales.* Both orders contain typical iron-oxidizing Bacteria such as *Leptothrix* (*Burkholderiales*), *Gallionella* and *Sideroxydans* (*Nitrosomonadales*) species. The presence of extracellular structures typical of *Leptothrix* species and *Gallionella* species was confirmed by electron microscopy (Figure S3 in Supporting Information S1).

Comparison of the surface and depth samples revealed significant differences in community composition hinting at a phylogenetic and functional stratification within the mat (t-test, *p*<0.02) (Figure 1b). While *Methylococcales* (*Gammaproteobacteria*) sequences were more abundant in the deeper samples, *Burkholderiales* (*Betaproteobacteria*) sequences dominated the surface samples. Conversely, *Nitrosomonadales* (*Betaproteobacteria*) sequences were more abundant in deep samples with 3.6% of the sequences compared to 0.3% at the surface.

### rRNA transcript-based diversity and richness analysis

**Figure 2:**
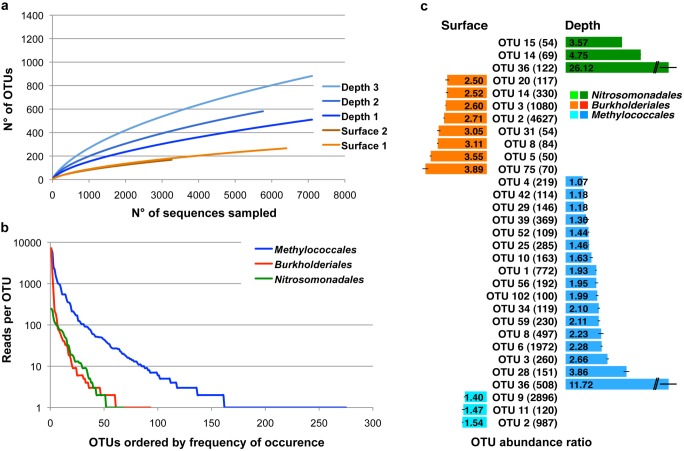
Estimation of the bacterial community diversity. Ribosomal RNA reads covering the variable 16S rRNA gene regions V3, V4 and V5 (393–925 *E. coli* positions) were analyzed on 462 positions (56.600 sequences). (a) Rarefaction curves for all five samples with combined replicates. For illustration purposes the curves were shorted to 7.000 sequences sampled. (b) Rank abundance distribution of OTUs from *Methylococcales*, *Burkholderiales* and *Nitrosomonadales*. All five samples and replicates were combined. (c) OTU abundance ratio of *Methylococcales*, *Burkholderiales* and *Nitrosomonadales*. Parentheses: Number of sequences included. Error bars: standard deviation. *P*-value from Wilcoxon signed rank test for each OTU group comparing surface versus depth: *Nitrosomonadales*: *p*<10^−5^, *Burkholderiales p*<10^−8^, *Methylococcales p*<10^−7^.

Our aim was to determine the diversity of the microbial community based on expressed rRNA gene sequences. Unlike amplicon sequences, metatranscriptomic sequences as produced herein are not biased by the choice of primers and the amplification steps. Analysis of the eukaryotic members of the microbial mat was not included due to the limited number of 18S and 28S rRNA gene sequences. We first determined the positions of the rRNA transcript fragments on the 16S rRNA genes. SSU rRNA reads which covered the nucleotide positions 393 to 925, including the V3, V4 and V5 variable regions (*E. coli* numbering), were selected and analyzed as described in the developed bioinformatics protocol (see Materials and Methods). The delineation of OTU and computation of richness estimates (Chao1 non parametric estimator) showed that the richness in surface samples was much lower than in samples collected at depth (Figure 2a). Alpha-diversity indexes, such as the Shannon and reciprocal Simpson indexes, revealed the same tendencies with lower values for the surface samples than for the depth samples (Table S2 in Supporting Information S1). A high number of OTUs were affiliated to *Burkholderiales*, *Nitrosomonadales* and *Methylococcales,* accounting for 84.5% on average of the sequences compared over the five samples (Figure S4 in Supporting Information S1). *Methylococcales* featured a much higher OTU richness (275 OTUs) than *Nitrosomonadales* (68 OTUs) and *Burkholderiales* (93 OTUs) (Figure 2b). Rank-abundance curves displayed strong negative slopes, thus revealing a low evenness in all three orders with a more pronounced effect in *Burkholderiales.* This indicated the dominance of a small number of OTUs in our dataset, particularly within the *Burkholderiales*.

In order to identify potential habitat specialization, we analyzed the depth/surface OTU distribution within the *Methylococcales, Burkholderiales* and *Nitrosomonadales,* respectively. Therefore we determined the number of sequences for each OTU present in each replicate of the two sample sites using surface and subjacent depth samples independently (Surface 1+2, Depth 1+2). OTUs constituted of at least 100 sequences for *Methylococcales* and 50 sequences for *Burkholderiales* and *Nitrosomonadales*, respectively, were retained for the relative OTU ratio calculation according the surface/depth distribution (Figure 2c). OTUs affiliated to *Burkholderiales* were significantly overrepresented in the surface samples ranging from 2.5−3.89 folds relative abundance compared to subjacent depth samples (Wilcoxon-test, *p*<10^−8^). On the contrary OTUs affiliated to *Nitrosomonadales* were strongly overrepresented in depth samples with phylotype ratios ranging from 3.57 to 26.12 (Wilcoxon-test, *p*<10^−5^). From a total of 20 OTUs in the *Methylococcales* group seventeen were overrepresented in the depth samples with phylotype ratios ranging from 1.07–11.72 while three showed slightly higher presence in the surface samples (1.40–1.54) (Wilcoxon-test, *p*<10^−7^).

### Linkage of community diversity to metabolic function

**Figure 3:**
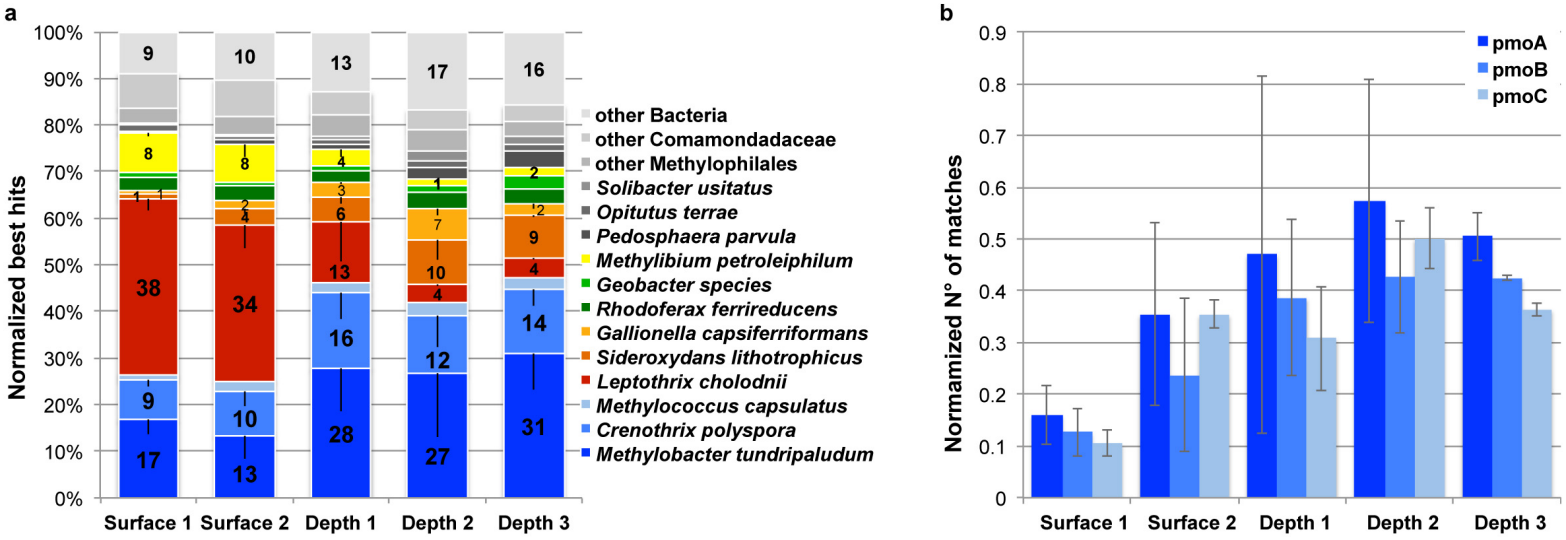
Linkage of community diversity to metabolic function. (a) Relative abundance of mRNA reads matching selected genomes from bacteria with relevant characteristic metabolisms: iron oxidation (red), methanotrophy (blue) and anaerobic iron respiration (green). (b) Relative abundance of *pmo*CAB matching transcripts coding for the three subunits of the methane monooxygenase. Error bars correspond to standard deviation from the replicates.

Analysis of the rRNA reads suggested that methanotrophs affiliated to *Methylococcales* and iron-oxidizers affiliated to *Burkholderiales* and *Nitrosomonadales* have distinct positions within the mat leading to a vertical functional differentiation. To strengthen this hypothesis, we analyzed reads of non ribosomal RNA transcripts by quantitative genome recruitment using a set of 39 bacterial reference genomes that showed the highest number of matches in BLAST analyses (Table S3 in Supporting Information S1). Most of these genomes are representative of particular metabolisms, notably methanotrophy, iron oxidation and iron reduction (Figure 3a). To ensure a reliable recruitment of transcript sequences, we applied very strict alignment and best match criteria (50% aa identity, 50% coverage). Under these conditions 22,671 sequences were recruited by the 39 bacterial genomes. Methanotrophs recruited most sequences from the deep (D) samples ranging from 41.3% to 46.0% of the matching reads with most matches to the genome of the type I methanotroph *Methylobacter tundripaludum* [20] whereas in surface (S) samples they represented only 25.0–26.5% (t-test, *p* = 0.004). Iron reducers recruited 3.5%–6.1% of the reads with most matches to the *Rhodoferax ferrireducens* genome. Conversely, 33.6–37.8% of the reads matched the genome of the iron-oxidizer *Leptothrix cholodnii* in surface samples but only 12.9% (D1), 4.1% (D2) and 4.4% (D3) in deep samples (t-test, *p*<10^−2^). Interestingly, the proportions of reads matching *Sideroxydans lithotrophicus* and *Gallionella capsiferriformans* genomes, both iron-oxidizing *Nitrosomonadales*, were significantly higher in deep samples than in surface samples, attaining 16.2% in D2 (t-test, *p* = 0.006).

Three genes from *Sideroxydans lithotrophicus*, *mto*A, *mto*B and *cyam*A were recently linked to iron oxidation [21]. In total, 157 sequences of the metatranscriptomes matched to the three genes (Figure S5 in Supporting Information S1). The relatively low number of matches and high variations between the samples did not allow detailed statistical distribution analysis. Nevertheless, transcripts coding for the (FeII)-oxidizing protein MtoA were identified in all 11 metatranscriptomes and relative abundance analysis of the three genes showed highest levels in D2 that correlates with the genome recruitment analysis were *Sideroxydans lithotrophicus* recruited 10% of the sequences.

### Expression of methane monooxygenase genes

To confirm the role of methanotrophs in the functioning of the mat community, we analyzed the expression patterns of the genes coding for the three subunits of the particulate methane monooxygenase (*pmo*CAB, Figure 3b) which is involved in the conversion of methane to methanol during the first step of methane oxidation [22]. Transcript analysis of these genes provides a good indication for methane oxidation activity level since *pmo*A transcript levels correlated well with whole-cell methane oxidation rates and cell growth as shown in *Methylosinus trichosporium* [23]. Under strict best match count conditions (60% aa identity, 50% coverage) 2,343 sequences matched *pmo*CAB genes affiliated to the type I methanotrophs *Methylococcales*, representing by far the most expressed protein-encoding genes in our sequence datasets. The second most abundant transcript, encoding a multidrug efflux pump (COG0841), was represented by 1210 sequences. Only 18 sequences matched to the soluble methane monooxygenase (sMMO) while no matches to other methane monooxygenases from *Crenothrix polyspora* (*Methylococcales*), *Methylacidiphilum fumariolicum* (*Verrucomicrobia*) and *Methylomirabilis oxyfera* (candidate phylum NC10) were detected. Representative relative values were obtained by normalizing the number of matches to the respective number of prokaryotic rRNA matches (see Materials and Methods). Although the analyzed triplicates showed strong variation, the relative abundance analysis revealed significantly higher levels of *pmo*CAB reads in the deep samples (t-test, *p* = 0.038).

## Discussion

To decipher the roles of microbial communities in ecosystem functioning, the fine identification of microbial taxa and processes is critical. In the present study, we report the analysis of expressed genes in an iron-rich microbial mat. Taxonomic and functional information carried by these genes provided valuable insights into the structure and activities of the microbial mat community. Numerous studies have evidenced the importance of typical neutrophilic iron oxidizers such as *Leptothrix* in the development of these microbial mats [6] as well as the presence of a high number of other microbial members [7]. However, no community gene expression profile was available for these mats.

The observed abundances of sequences in our metatranscriptomes resulted from combined effects of many parameters among which population size, gene copy number per genome, specific growth rate, metabolic state and life history. None of these parameters are readily accessible in natural communities. In spite of these potential caveats, random sequencing of the total ribosomal RNA offers a powerful and straightforward way to infer community structure, as it does not suffer of the bias associated with the choice of primers for PCR-based methods and RNA depletion methods [24]. Furthermore, it has the potential to detect more easily low-abundant organisms as every cell, whatever their metabolic state, are likely to contain more rRNA than rRNA genes. Metatranscriptome sequencing also informs on the nature of the *in-situ* expressed functions, thus providing a more direct view of the nature of the metabolic processes carried out by the community.

In consequence, the approach used in this study and others allows capturing a more faithful picture of the microbial community [11]–[13]. The observed similarity between the analyzed triplicates bears testimony to the reliability of the applied strategy.

### Putative interaction between iron oxidizers and methanotrophs

Our data showed that the major bacterial players responsible for the construction and functioning of the microbial mat included, as expected, FeOB affiliated to *Leptothrix* (*Burkholderiales*) and, to a lesser extent, *Gallionella* and *Sideroxydans* (*Nitrosomonadales*). Surprisingly, high proportions of type I methanotrophs (*Methylococcales*) rRNA sequences were observed in our data set. Applying PCR based methods, the presence of potential methane-oxidizing bacteria (MOB) and iron-oxidizers have already been reported in similar iron-rich microbial mats [10], in riparian wetlands [25] and in deep-sea fields [26]–[29]. We provide the first community expression data consistent with the presence of an abundant and diverse guild of methane-oxidizing bacteria in an iron-rich mat built by neutrophilic FeOB.

Our results strongly suggest that iron-oxidation and methane-oxidation occur concomitantly in the mat. MOBs and the majority of FeOBs identified in the present study have an obligatory aerobic metabolism. Therefore, they are typically observed at oxic-anoxic interfaces where they find both oxygen and reduced electron donors. Iron oxidation is thermodynamically more favorable and yields more energy than methane oxidation in oxygen-depleted environments. The neat stratification highlighted in the mat with MOBs outnumbering FeOBs in the depth layer brings insight into the respective ecology of the two groups of microorganisms. Detailed oxygen profiles measured *in situ* in similar microbial mats showed decreasing oxygen concentrations with depth [7],[30]. We showed significantly higher methane concentrations in the presence of the microbial mat. Moreover iron-oxidation by *Leptothrix* species in the surface layer should reduce the availability of oxygen for microorganisms living in the depth layer. In contrast to *Leptothrix*, *Gallionella* and *Sideroxydans* show distribution patterns similar to those of methanotrophs. *Gallionella* has been shown to grow in presumably more oxygen-limiting conditions [1],[2]. Thus, *Gallionella* and methanotrophs might share similar environmental requirements, especially with regards to oxygen concentration. Based on these observations, one can speculate that competition for oxygen and the availability of methane could drive the distribution of FeOB and MOB in the mat. Methanotrophs would be able to efficiently outcompete *Leptothrix* at depth where the counter gradient of methane and oxygen would be more favorable for MOB.

### Methane cycling and diversity of methanotrophs

Ribosomal RNA reads affiliated to methanogens as well as sequences of genes encoding for the key enzyme of methanogens methyl-coenzyme M reductase were found exclusively in depth samples. This is consistent with the fact that methanogens require a reduced environment to produce methane. However, few sequences of methanogens were detected indicating that these microorganisms are only a minor component of the mat community. These observations do not support the hypothesis of methane production within the mat but the existence of a flux of methane produced in the permanently flooded, organic-rich sediments below the mat where methanogens are the most likely to thrive.

Methane-oxidizing Proteobacteria have been subdivided in three groups (type I, X and II) on the basis of phylogenetic reconstructions using genes encoding the 16S rRNA gene and the beta subunit of the particulate monooxygenase (*pmo*A) as well as on differences in carbon fixation metabolism, lipid membrane composition and cellular ultrastructure [31]. Type I and type X methanotrophs belong to *Gammaproteobacteria* while type II are members of the *Alphaproteobacteria*. Interestingly, almost all sequences of methanotrophs residing in the mat were affiliated to type I methanotrophs. These have been found to dominate in high-methane environments such as lake sediments [32], wetland and landfill soils [33] where they carried methane oxidation. In our analysis the methane concentrations were mostly higher in deep samples. This strengthens the hypothesis of a significant efflux of methane circulating through the mat. Albeit precise measurements of methane consumption remain to be done, the strong presence of methanotrophs observed here indicates that the iron-rich microbial mat could represent a to-date undescribed player involved in methane cycle.

Remarkably, *Methylococcales* showed a significantly higher richness and evenness indicating that a large number of *Methylococcales* OTUs were involved in methane oxidation. Methanotrophs consume methane to obtain carbon and energy. The identical metabolic activities and similar substrate requirements could induce competition for methane and oxygen between OTUs affiliated to MOB. The OTU ratio distribution analysis hinted strongly to environmental specialization of type I MOBs. Similarly, the adaptation of OTUs from type I MOBs to different concentrations of methane and oxygen along a depth profile has been demonstrated in a flooded paddy soil [34]. Hence, a likely explanation for such a level of diversity for the methanotrophs residing in the mat is the coexistence of numerous microenvironments defined by the methane and oxygen gradients.

### Iron redox cycling in the mat

Besides strong expression levels of genes indicative of iron-oxidizers and methanotrophs, we showed the presence of iron-reducing bacteria, thus confirming at the molecular level the existence of an iron redox cycle in the mat. This is in agreement with recent *in situ* microvoltametric determinations of Fe(II), Fe(III) and O_2_ concentrations in a comparable microbial mat which suggested iron redox cycling [7]. Iron reducing bacteria only accounted for 1.26% of the bacterial OTUs and 2.85% of the SSU rRNA sequences on average. Likewise genome sequences of known iron reducing bacteria recruited a small proportion of mRNA reads. This is comparable to recent studies where iron reducers were estimated to account for a minor proportion of the community using 16S rRNA gene amplicon sequencing [6],[7]. Thus, iron-reducers contributed little to community-expressed functions in the mat at the time of sampling.

## Conclusion

By analyzing 14 metatranscriptomes, we raise new detailed information of the composition, diversity and the metabolic processes of the microbial denizens in a typical iron-rich microbial mat. We provide strong evidences that, despite a high taxonomic richness, a small number of taxa take over the engineering and the functioning of the mat. Despite the fragile and heterogeneous nature of the mat, we confirm our working hypothesis, the existence of a vertical stratification of the mat community deduced from the expression patterns of phylogenetic and functional marker genes. Our results suggest that, beside the iron-oxidizing bacteria, significant populations of methane oxidizing bacteria are involved in the functioning of the microbial mat ecosystem. Linking community gene expression patterns with detailed measurements of iron and methane fluxes will help untangling the complex interplay between microbial diversity and biogeochemical functioning in the mat.

## Materials and Methods

### Sampling procedure

**Figure 4:**
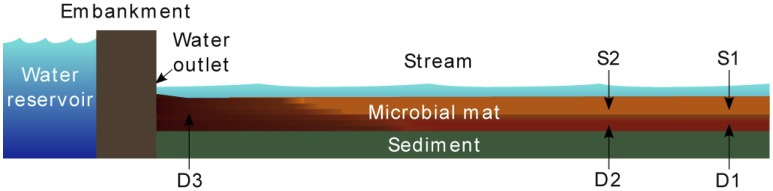
Schematic profile of the iron rich microbial mat with sampling sites.

The sampling site is a shallow, slow flowing stream where the mat is constantly present. The stream is fed by continuous seepages of ferrous-rich anoxic water at the base of the dam of a small reservoir in the Brocéliande forest. The bottom of the streambed consists of a layer of dead leaves and organic-rich sediments over which the mat grows. Microbial mat samples were collected at three locations (Figure 4) separated by a distance of about 2 meters. For locations 1 and 2, samples were harvested at a depth of 1 cm below the water surface in the orange flocculent layer (S1, S2) and at 7–9 cm below the water surface in the depth layer (D1, D2) on June 8, 2010. Location 3 was situated near the water outlet and samples were collected only in the depth layer as no characteristic surface layer was observed at the time of sampling (D3). No specific permissions were required to sample at these locations (Brittany, France; 48.00096°N, −2.27727°W) and no endangered or protected species were involved. Samples of 50 ml were collected in sterile polypropylene tubes. Aliquots of 2 ml were prepared as quickly as possible, spun at 15,000 g for 4 min and pellets were frozen in liquid nitrogen and stored at −80°C until processing. In all, 15 samples were collected and submitted to RNA extraction and metatranscriptomic analysis. All samples were prepared independently to avoid cross contaminations. Environmental parameters were directly measured in the field at each sampled site and depth using specific probes: temperature and pH (Sentix 41, WTW), dissolved oxygen concentration (Pioneer-20) and redox potential (501-Redox-Mettler-Toledo, InLab). The dissolved inorganic carbon (DIC) and dissolved organic carbon (DOC) concentrations as well as iron concentrations were determined after filtration at 0.2 µm. DIC and DOC were determined by thermic oxidation (Shimadzu-TOC-5050A analyzer). Iron concentrations (Fe^2+^, Fe^3+^) were quantified using the 1,10-phenanthroline spectrophotometry method [35] (Table S1 in Supporting Information S1). For methane concentration measurements samples were collected in 250 ml glass bottles immersed in water. The CH_4_ concentrations were determined by headspace extraction with helium as host gas and analyzed by gas chromatography with a thermal conductivity detector (GC/TCD, Agilent MicroGC 3000A).

### Preparation of cDNA libraries for pyrosequencing

We obtained 1–3 µg of total RNA and 70–300 ng of cDNA from about 600 mg of centrifuged microbial mat. Metatranscriptomic libraries were prepared from each of the 15 samples. RNA extraction was performed following a newly designed protocol to maximize the yield and quality allowing direct sequencing without any amplification step. Cells stored as pellets in 2 ml tubes were lysed in RNA extraction buffer containing 4% cetyltrimethylammonium bromide (CTAB), 0.5% polyvinylpyrrolidone (PVP, Sigma-Aldrich), 0.7 M NaCl, 100 mM potassium phosphate (pH 6.8), 20 mM EDTA (pH 8.0), 1% beta-mercaptoethanol, 1 M guanidin thiocyanate at 65°C for 10 min and homogenized in matrix lysis tubes E (MP-Biomedicals) for 1 min (Retsch-Mixer-Mill-MM400, Germany). One volume of chloroform-isoamylalcohol (24∶1) was added, mixed for 1 min and incubated at room temperature for 5 min. The samples were centrifuged at 12,000 g for 10 min at 4°C and the aqueous phases were transferred to new tubes. Binding conditions for silica-based RNA extraction were adjusted, applied on Nucleo Spin RNA II kit columns and subsequent purification and DNase treatment was performed following the instructions of the manufacturer (Macherey-Nagel). Fragmentation of total RNA was performed as indicated in the cDNA Rapid Library Preparation Method Manual (454/Roche) by applying 0.1 volumes of the ZnCl_2_ fragmentation solution (90 mM ZnCl_2_; 100 mM Tris-HCl, pH 7.0) for 85 s at 70°C. The absence of remaining DNA was confirmed by PCR using *pmo*A gene specific primers (A189F-mb661R) and 16S rRNA gene primers (U27F-U1492R). Double-stranded cDNA generation was performed with the Universal RiboClone cDNA Synthesis System (Promega). The cDNA was purified with Agencourt AMPur XP magnetic beads (Beckman-Coulter). RNA and cDNA quality was checked with the RNA Pico 6000 Pico kit or the High sensitivity DNA kit on a 2100 Bioanalyzer (Agilent). Subsequent library construction and pyrosequencing was performed in technical duplicates on a GS FLX system (454/Roche) at the “Functional and Environmental Genomics” platform (OSUR, Rennes, France). Roche/454 filtering tools and stringent filters developed locally were used to ensure the highest possible sequence quality and to suppress artificial replicates of sequences as well as sequences smaller than 250 bp. From a total of 2,776,339 reads 2,082,630 were used for subsequent analysis. One replicate of the site D3 failed in the sequencing and was excluded (Table 1).

### Diversity and richness estimation

For taxonomic assignment in the relative abundance analysis, rRNA encoding sequences were identified by performing BLASTs against the LSU and the SSU rRNA SILVA non-redundant reference database (104) with a stringent e-value cut off (e-40) [17] and interpreted with MEGAN [18]. Each replicate was treated separately. The best matches were counted and normalized to the total number of SSU or LSU rRNA matches respectively.

For diversity and richness estimations SSU and LSU rRNA matching sequences were identified using Meta_RNA [36] after slight modification of the script (hmm_rRNA3.py) to allow printing of the matching positions of the sequences on the reference Hidden-Markov-Models. Parsing the output of the Meta_RNA script identified highly populated rRNA regions. Sequences that covered the V3, V4 and V5 variable SSU rRNA region (393–925 *E. coli* position) were selected for subsequent analysis. In total 56600 sequences from the 5 samples matching this region were included in the analysis. The sequences were aligned with SINA aligner [17], imported into ARB [37], and sequences were trimmed to start and stop at the same position in the reference alignment (*E. coli* position 393–925). Dereplication was obtained with the “unique.seqs” command of the MOTHUR package [38] (Mothur 1.20.3). The distance matrices for each sample (3 triplicates combined) were calculated with ESPRIT_PC (−f −r options) that uses a pairwise alignment approach [39]. A MOTHUR-compatible distance matrix was obtained with an in-house perlscript. MOTHUR was used for clustering (command “cluster” with “furthest neighbour” option) and the “rarefaction.single” and “summary.single” commands were used to calculate diversity and richness indices. To separate the results in the different samples or replicates the commands “make.shared” and “summary.single” were used with the “group” option. For taxonomic affiliation and rank abundance analysis “classify.seqs” and “classify.otu” were used with the “silva.bacteria” taxonomy (up to the order level with 100% confidence value).

### Genome recruitment

The transcript recruitment analysis was performed using the PROMER package included in the MUMmer 3.22 sequence alignment software tool [40] essentially as described previously [41]. Best matching transcript sequences in preliminary NCBI nr BLAST (e-10) were aligned to a set of 39 reference genomes using PROMER under standard conditions. These genomes were chosen because they were the most represented in the best matches of transcripts in the NCBI nr database. Since the genome of *Crenothrix polyspora* is not assembled the available contigs were concatenated prior to the analysis. SHOW-COORDS was used to parse the output file using different options (−k, −l, −r). Sequences with less than 50% coverage and 50% identity were eliminated and only the best matches were retained. The numbers of matches to each genome were counted for each triplicate and normalized to the total number of respective matches. The average values from the replicates are shown for the five samples (Figure 3).

### Quantitative gene expression analysis

A database consisting of full-length and near full-length genes encoding for the three methane monooxygenase subunits *pmo*CAB was constructed. Non-rRNA transcripts were aligned to the pmo_db using the PROMER program included in the MUMmer package (3.22). The delta file was parsed with the SHOW-COORDS program applying –c –r options. Cut-off for match count was set at 60% amino acid identity and 50% read coverage. Match counts from replicates were normalized to the *pmo*CAB gene lengths and to the total number of prokaryotic rRNA gene-matching transcripts, respectively. For *mtr*A, *mtr*B and *cym*A transcript analysis the reads matching the corresponding genes from *Sideroxydans lithotrophicus* ES-1 were counted and processed in the same manner.

### Statistical analysis

Statistical analysis was performed using the parametric t-test as well as the non-parametric Wilcoxon test implemented in R [42] (2010). The standard errors of means were calculated and used to estimate the variability of each parameter. Non-parametric Wilcoxon-test was used to detect significant differences between depths or stations as indicated.

### Data deposition

Pyrosequencing reads reported in this publication have been deposited in the Sequence Read Archive under the study accession number PRJEB5474.

## Supporting Information

Supporting Information S1 **File contains Figures S1–S5 and Tables S1–S3. Figure S1:** Methane concentrations measured at the sampling sites. Methane concentrations were measured at the two sampling sites (U, upstream; D, downstream) in triplicates distinguishing between depth and surface. For two samples (Surface Jan 2013, D; Surface May 2013, D) only a single measurement was available. Higher methane concentrations were measured in corresponding deep samples with one exception in the samples July 2013, U. Surrounding waters without microbial mat observation are shown in grey: Source, water flow directly upstream of the microbial mats; Lake, lake above the stream without microbial mat; River, main stream with stronger water current. **Figure S2:** Relative abundance of the active eukaryotic members of the microbial community based on the taxonomic affiliation of SSU rRNA transcript matches at the phylum and class levels. The rRNA transcripts from each triplicate were combined and normalized average values are shown. The total numbers of eukaryotic matches included in the analysis are indicated. The large majority of eukaryotic matches are affiliated to *Tetrahymenina*/*Oligohymenophorea* that contains essentially freshwater grazers such as *Tetrahymena* species (ranging from 62.3% to 91.0% of the eukaryotic matches). **Figure S3:** Scanning electron microscopy of the microbial mat showing typical extracellular sheets (*Leptothrix*) and stalks (*Gallionella*). **Figure S4:** Relative abundance and taxonomic affiliations of OTUs. For illustration purposes standard deviations are indicated as negative or positive bars. **Figure S5:** Relative abundance of reads matching transcripts coding for the three proteins involved in iron oxidation from *Sideroxidans lithotrophicus* ES-1. *Mtr*A, *mtr*B, *cym*A transcripts from each triplicate were combined and normalized average values are shown (in total 157 matches). **Table S1:** General characteristics of the microbial mat samples. **Table S2:** Diversity and richness estimates for the rRNA region analysis. **Table S3:** Detailed match counts of non-rRNA transcripts to microbial genome sequences.
(PDF)
