# Supplementary material for: Unraveling the Stratification of an Iron-Oxidizing Microbial Mat by Metatranscriptomics
Source: PLoS One. 2014 Jul 17;9(7):e102561. doi: 10.1371/journal.pone.0102561 (PMC4102501; doi:10.1371/journal.pone.0102561)
Supplement: Supporting Information S1 — File contains Figures S1–S5 and Tables S1–S3. Figure S1: Methane concentrations measured at the sampling sites. Methane concentrations were measured at the two sampling sites (U, upstream; D, downstream) in triplicates distinguishing between depth and surface. For two samples (Surface Jan 2013, D; Surface May 2013, D) only a single measurement was available. Higher methane concentrations were measured in corresponding deep samples with one exception in the samples July 2013, U. Surrounding waters without microbial mat observation are shown in grey: Source, water flow directly upstream of the microbial mats; Lake, lake above the stream without microbial mat; River, main stream with stronger water current. Figure S2: Relative abundance of the active eukaryotic members of the microbial community based on the taxonomic affiliation of SSU rRNA transcript matches at the phylum and class levels. The rRNA transcripts from each triplicate were combined and normalized average values are shown. The total numbers of eukaryotic matches included in the analysis are indicated. The large majority of eukaryotic matches are affiliated to Tetrahymenina/Oligohymenophorea that contains essentially freshwater grazers such as Tetrahymena species (ranging from 62.3% to 91.0% of the eukaryotic matches). Figure S3: Scanning electron microscopy of the microbial mat showing typical extracellular sheets (Leptothrix) and stalks (Gallionella). Figure S4: Relative abundance and taxonomic affiliations of OTUs. For illustration purposes standard deviations are indicated as negative or positive bars. Figure S5: Relative abundance of reads matching transcripts coding for the three proteins involved in iron oxidation from Sideroxidans lithotrophicus ES-1. MtrA, mtrB, cymA transcripts from each triplicate were combined and normalized average values are shown (in total 157 matches). Table S1: General characteristics of the microbial mat samples. Table S2: Diversity and richness estimates for the rRNA reg [file pone.0102561.s001.pdf]

Quaiser et al.

Supporting Information

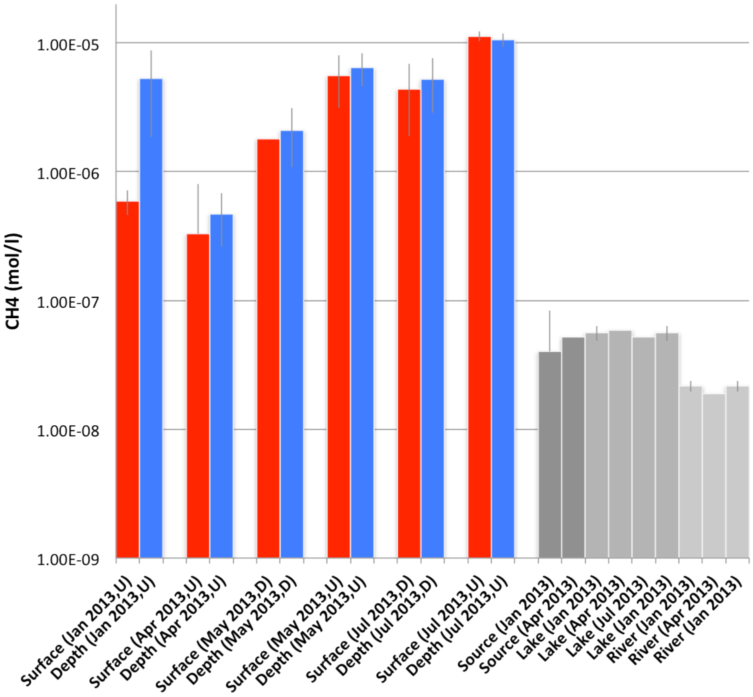

Figure S1. Methane concentrations measured at the sampling sites. Methane concentrations were measured at the two sampling sites (U, upstream; D, downstream) in triplicates distinguishing between depth and surface. For two samples (Surface Jan 2013,D; Surface May 2013,D) only a single measurement was available. Higher methane concentrations were measured in corresponding deep samples with one exception in the samples July 2013, U. Surrounding waters without microbial mat observation are shown in grey: Source, water flow directly upstream of the microbial mats; Lake, lake above the stream without microbial mat; River, main stream with stronger water current.

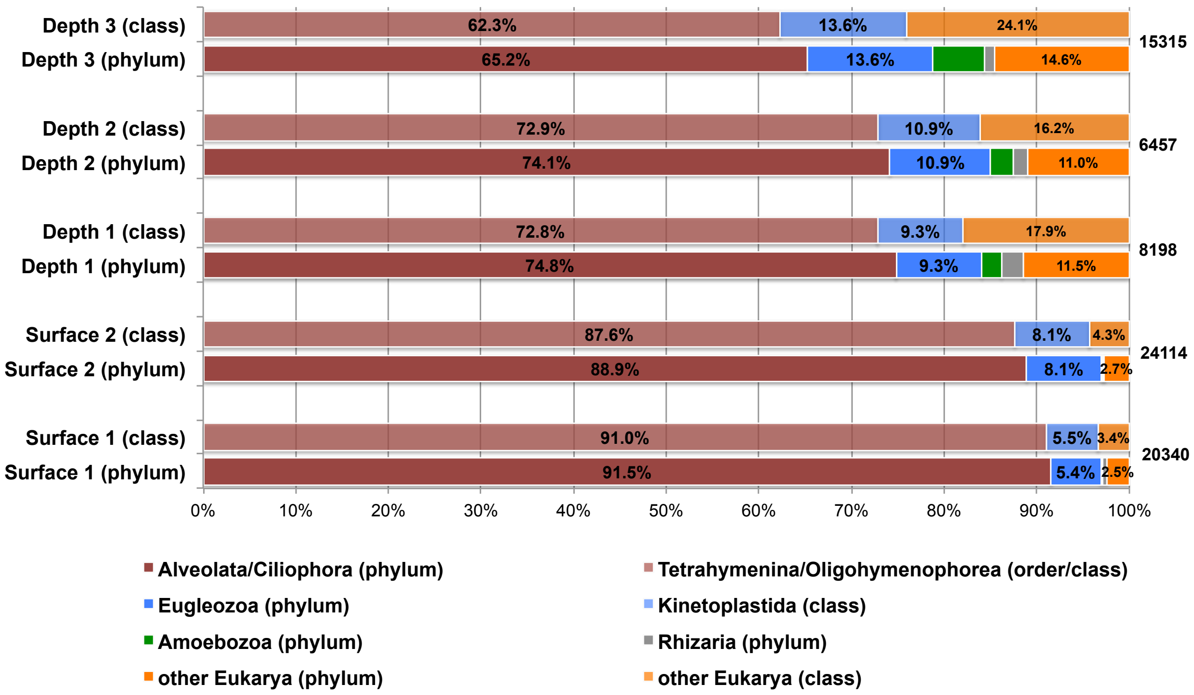

Figure S2. Relative abundance of the eukaryotic members of the microbial community based on the taxonomic affiliation of SSU rRNA read matches at the phylum and class levels. The rRNA reads from each triplicate were combined and normalized average values are shown. The total numbers of eukaryotic matches included in the analysis are indicated. The large majority of eukaryotic matches are affiliated to Tetrahymenina/Oligohymenophorea that contains essentially freshwater grazers such as *Tetrahymena* species (ranging from 62.3% to 91.0% of the eukaryotic matches).

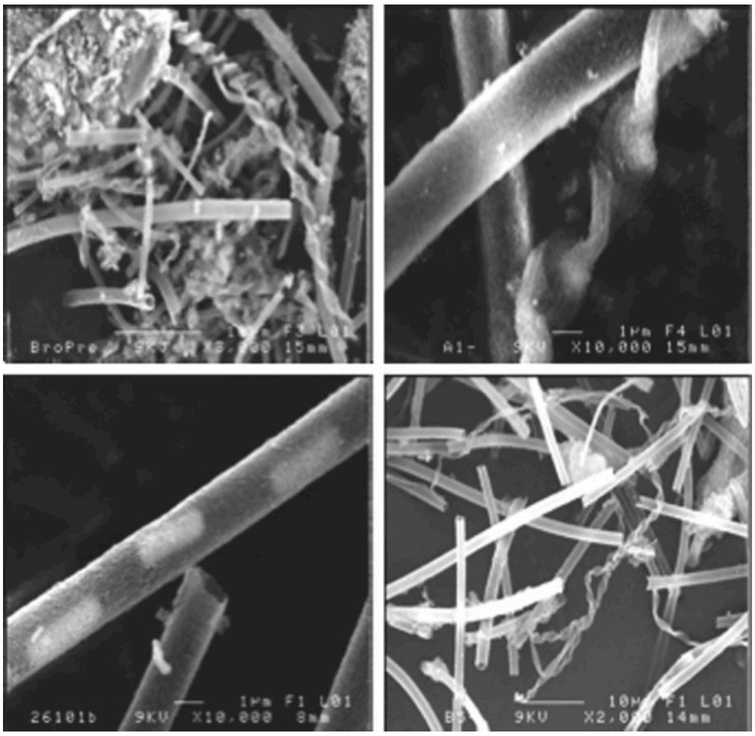

Figure S3. Scanning electron microscopy of the microbial mat showing typical extracellular sheets (*Leptothrix*) and stalks (*Gallionella*).

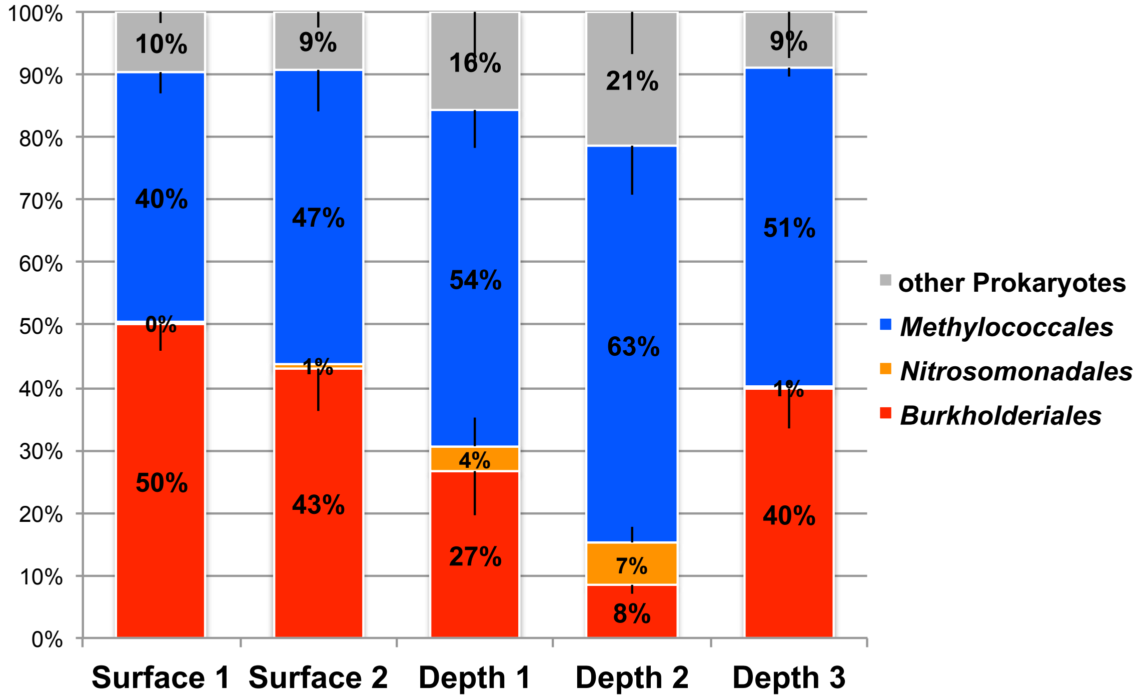

Figure S4. Relative abundance and taxonomic affiliations of OTUs. For illustration purposes confidence intervals from the triplicates are indicated either as negative or positive bars.

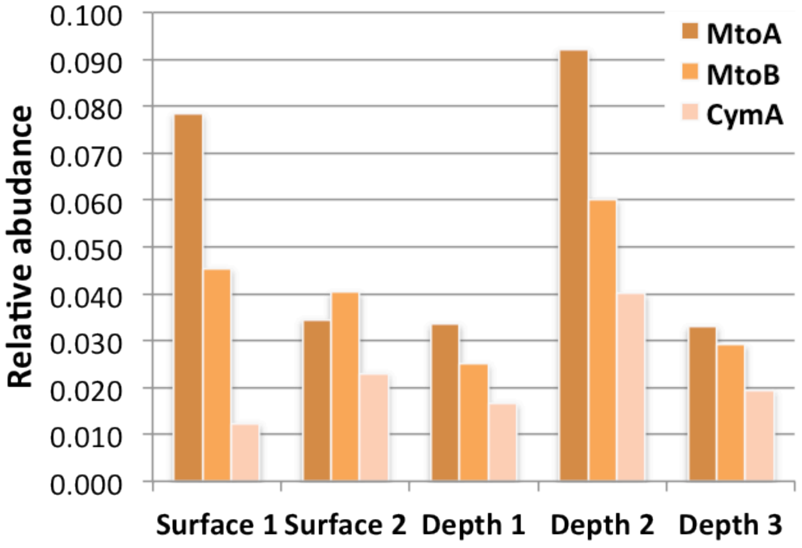

Figure S5. Relative abundance of reads matching transcripts encoding the three proteins involved in iron oxidation from *Sideroxidans lithotrophicus* ES-1. *Mtr*A, *mtr*B, *cym*A transcripts from each triplicate were combined and normalized average values are shown (in total 158 matches).

Table S1. General characteristics of the microbial mat samples.

	Surface 1 (S1)	Surface 2 (S2)	Depth 1 (D1)	Depth 2 (D2)	Depth 3 (D3)
Sampling date	08.07.2010	08.07.2010	08.07.2010	08.07.2010	08.07.2010
Aspect	Clear orange, Flocculent	Clear orange, Flocculent	Brownish-red, Flocculent with precipitates	Brownish-red, Flocculent with precipitates	Dark brown Slimy dense precipitates
Collection depth	Surface (1-3 cm) Above D1	Surface (1-3 cm) Above D2	Depth (6-8 cm) Below S1	Depth (6-8 cm) Below S2	Depth (on sed.*)
T (°C)	16.6 °C	15.0 °C	15.7 °C	15.4 °C	15.3 °C
pH	6.85	6.94	6.84	6.7	6.7
Dissolved oxygen (mg/l)	1.8	1.45	2.25	1.31	1.51
Redoxpotential (mV)	317	306	277	178	197
IC (mg/l)	2.17	1.96	1.98	1.96	1.91
DOC (mg/l)	10.38	10.51	11.77	8.76	10.81
Fe^{2+} (ppm or mg/l)	1.6	5.21	2.31	9.28	12.64
Fe^{3+} (ppm or mg/l)	0.11	0.12	0.14	0.32	0.1
Comment	triplicate	triplicate	triplicate	triplicate	Duplicate

* On sed.: thin mat in contact with surface and sediment

Table S2. Diversity and richness estimates for the rRNA region analysis.

	Surface 1 (S1)	Surface 2 (S2)	Depth 1 (D1)	Depth 2 (D2)	Depth 3 (D3)
			**Richness estimation**		
N° of OTUs	383	244	675	756	2208
Ace	1211	722	2443	2646	4941
Chao	798.34	543.09	1677.50	1569.40	3894.25
			**Diversity estimation**		
Shannon	2.87	2.95	4.10	4.81	4.83
Reciprocal Simpson	5.34	5.99	13.02	28.07	23.79
N° of reads included	6,392	3,254	7,089	5,749	34,116

Table S3. Detailed match counts of non rRNA transcripts to microbial genome sequences

N° of matches from species summarized in histogram (Fig. 3a)

Genomes used for best match identification	Taxonomic affiliation of species	summarized affiliation (if adequate)	S1_1	S1_2	S1_3	D1_1	D1_2	D1_3	D2_1	D2_2	D2_3	S2_1	S2_2	S2_3	D3_1	D3_2	sum of matches /genome
Methylovulum miyakonense mmoXplus (306921970)	Gammaproteobacteria,Methylococcales	other Bacteria	0	0	0	0	0	1	0	0	1	0	0	0	0	0	2
Methylovulum miyakonense orf5plus (306921963)	Gammaproteobacteria,Methylococcales	other Bacteria	2	11	3	9	49	3	22	18	1	5	3	1	59	26	212
Azoarcus (119896292)	Betaproteobacteria, Rhodocyclaceae	other Bacteria	5	4	8	1	1	14	0	7	18	5	1	4	13	0	81
Candidatus Nitrospira defluvii (302035394)	Nitrospireae	other Bacteria	0	2	5	1	1	3	1	7	48	1	1	1	11	1	83
Candidatus Koribacter versatilis (94967031)	Acidobacteria	other Bacteria	0	1	5	3	0	10	1	6	47	4	3	0	18	3	101
Anaeromyxobacter (153002879)	Deltaproteobacteria, Myxococcales	other Bacteria	1	2	11	0	2	12	3	9	42	2	4	6	20	5	119
Aromatoleum aromaticum (56475432)	Betaproteobacteria, Rhodocyclaceae	other Bacteria	0	6	12	3	2	14	1	11	38	2	2	5	20	4	120
Gemmatimonas aurantiaca (226225406)	Gemmatimonadetes	other Bacteria	4	2	21	4	2	19	1	8	41	1	1	2	21	1	128
Sorangium cellulosum (162448269)	Deltaproteobacteria, Myxococcales	other Bacteria	3	8	13	4	3	9	0	7	57	8	2	2	19	5	140
Thiobacillus denitrificans (74316018)	Betaproteobacteria, Hydrogenophilales	other Bacteria	2	7	16	1	4	22	0	9	46	1	5	9	14	4	140
Chitinophaga pinensis (256419057)	Bacteroidetes, Sphingobacteria	other Bacteria	0	7	10	3	8	12	0	8	40	2	6	1	36	8	141
Thauera sp. MZ1T (237653092)	Betaproteobacteria, Rhodocyclaceae	other Bacteria	7	11	23	2	1	20	0	3	28	17	3	4	21	4	144
Dechloromonas aromatica (71905642)	Betaproteobacteria, Rhodocyclaceae	other Bacteria	2	10	28	5	2	25	6	23	56	5	4	3	11	8	188
Anaerolinea thermophila (320159410)	Chloroflexi	other Bacteria	4	5	13	4	1	13	1	13	77	6	3	2	45	14	201
Bradyrhizobium japonicum (27375111)	Alphaproteobacteria, Rhizobiales	other Bacteria	3	2	4	3	8	21	3	24	95	3	3	6	37	8	220
Pelobacter propionicus (118578449)	Deltaproteobacteria, Desulfuromonadales	other Bacteria	0	8	35	3	0	23	0	4	57	5	3	9	64	10	221
Candidatus Accumulibacter phosphatis (257091663)	Betaproteobacteria, unclassified	other Bacteria	5	10	36	8	5	32	2	15	48	13	11	11	39	6	241
Chthoniobacter flavus (WGS NZ_ABVL01000000)	Verrucomicrobia	other Bacteria	1	9	20	5	9	23	0	18	81	5	7	3	59	15	255
Variovorax paradoxus (319790694)	Burkholderiales, Comamonadaceae	other Comamonadaceae	9	19	41	4	0	29	1	4	33	13	4	6	25	7	195
Polaromonas naphthalenivorans CJ2 plasmids	Burkholderiales, Comamonadaceae	other Comamonadaceae	0	2	2	1	0	1	0	2	3	6	2	3	4	1	27
Polaromonas naphthalenivorans CJ2 chrom (121602919)	Burkholderiales, Comamonadaceae	other Comamonadaceae	4	12	35	9	1	33	0	10	60	16	7	7	26	5	225
Polaromonas sp. JS666 (91785913)	Burkholderiales, Comamonadaceae	other Comamonadaceae	8	13	58	4	2	42	0	13	61	9	11	14	33	6	274
Curvibacter putative symbiont (260219428)	Burkholderiales, Comamonadaceae	other Comamonadaceae	9	26	70	7	4	47	3	24	99	27	15	17	38	8	394
Acidovorax_delafieldii (WGS NZ_ACQT01000000)	Burkholderiales, Comamonadaceae	other Comamonadaceae	10	19	65	17	4	48	1	8	63	15	10	16	23	7	306
Methylobacillus flagellatus (91774356)	Betaproteobacteria; Methylophilales	other Methylophylales	0	3	18	5	2	24	0	10	30	6	4	4	12	6	124
Methylovorus sp. MP688 (313199777)	Betaproteobacteria; Methylophilales	other Methylophylales	3	5	10	7	2	21	1	15	50	8	2	1	12	6	143
Methylotenera mobilis (253995374)	Betaproteobacteria; Methylophilales	other Methylophylales	1	9	21	7	3	39	3	10	49	6	4	14	21	10	197
Methylotenera versatilis (297537197)	Betaproteobacteria; Methylophilales	other Methylophylales	10	17	89	13	8	110	2	31	170	8	21	10	50	17	556
Geobacter metallireducens (78221228)	Deltaproteobacteria, Desulfuromonadales	Geobacter species	0	2	16	0	3	4	1	3	15	0	2	0	21	6	73
Geobacter lovleyi (189423082)	Deltaproteobacteria, Desulfuromonadales	Geobacter species	3	0	22	5	0	14	1	9	27	1	1	1	43	8	135
Geobacter uraniireducens (148262085)	Deltaproteobacteria, Desulfuromonadales	Geobacter species	1	3	17	3	2	20	1	7	36	3	4	2	46	13	158

N° of matches from species represented in histogram (Fig. 3a)

Genomes used for best match identification	Taxonomic affiliation of species	S1_1	S1_2	S1_3	D1_1	D1_2	D1_3	D2_1	D2_2	D2_3	S2_1	S2_2	S2_3	D3_1	D3_2	sum of matches /genome
**other Bacteria**		39	105	263	59	98	276	41	190	821	85	62	69	507	122	2737
**other Comamondadaceae**		40	91	271	42	11	200	5	61	319	86	49	63	149	34	1421
**other Methylophilales**		14	34	138	32	15	194	6	66	299	28	31	29	95	39	1020
**Solibacter usitatus (116222307)**	Acidobacteria	3	4	28	6	2	35	2	27	147	4	3	0	64	15	340
**Opitutus terrae (182411826)**	Verrucomicrobia	10	3	45	1	3	54	0	21	129	10	2	6	88	7	379
**Pedosphaera parvula Ellin514 (WGS,ABOX00000000)**	Verrucomicrobia	3	5	25	5	2	70	2	29	258	14	9	2	189	19	632
**Methylibium petroleiphilum (124265193)**	Burkholderiales incertae sedis	50	88	303	29	10	152	3	10	120	84	35	76	53	17	1030
**Geobacter species**		4	5	55	8	5	38	3	19	78	4	7	3	110	27	366
**Rhodoferax ferrireducens T118 (89343559)**	Burkholderiales, Comamonadaceae	12	32	124	17	7	105	3	41	286	38	20	23	163	20	891
**Gallionella capsiferriformans (302580034)**	Gallionellaceae	4	8	34	20	19	87	16	84	383	13	12	12	110	18	820
**Sideroxydans lithotrophicus (291582584)**	Gallionellaceae	6	11	46	30	28	215	17	106	718	40	25	19	334	96	1691
**Leptothrix cholodnii (170774137)**	Burkholderiales incertae sedis	218	401	1376	90	25	639	15	41	210	280	200	312	147	51	4005
**Methylococcus capsulatus (66270661)**	Gammaproteobacteria, Methylococcales	5	12	33	9	15	64	8	31	125	13	16	17	85	26	459
**Crenothrix polyspora**	Gammaproteobacteria, Methylococcales	46	122	260	137	108	290	67	96	423	62	94	63	390	177	2335
**Methylobacter tundripaludum (344939781)**	Gammaproteobacteria, Methylococcales	93	226	479	188	207	564	138	247	810	71	131	107	887	397	4545
	sum of matches/replicate	547	1147	3480	673	555	2983	326	1069	5126	832	696	801	3371	1065	22671
